# 6-Shogaol induces apoptosis in human leukemia cells through a process involving caspase-mediated cleavage of eIF2α

**DOI:** 10.1186/1476-4598-12-135

**Published:** 2013-11-12

**Authors:** Qun Liu, Yong-Bo Peng, Ping Zhou, Lian-Wen Qi, Mu Zhang, Ning Gao, E-Hu Liu, Ping Li

**Affiliations:** 1 State Key Laboratory of Natural Medicines, China Pharmaceutical University, Nanjing 210009, China; 2 Department of Pharmacognosy, College of Pharmacy, 3rd Military Medical University, Chongqing 400038, China

**Keywords:** 6-shogaol, Leukemia, Apoptosis, Shotgun proteome, eIF2α

## Abstract

**Background:**

6-Shogaol is a promising antitumor agent isolated from dietary ginger (*Zingiber officinale*). However, little is known about the efficacy of 6-shogaol on leukemia cells. Here we investigated the underlying mechanism of 6-shogaol induced apoptosis in human leukemia cells *in vitro* and *in vivo*.

**Methods:**

Three leukemia cell lines and primary leukemia cells were used to investigate the apoptosis effect of 6-shogaol. A shotgun approach based on label-free proteome with LC-CHIP Q-TOF MS/MS was employed to identify the cellular targets of 6-shogaol and the differentially expressed proteins were analyzed by bioinformatics protocols.

**Results:**

The present study indicated that 6-shogaol selectively induced apoptosis in transformed and primary leukemia cells but not in normal cells. Eukaryotic translation initiation factor 2 alpha (eIF2α), a key regulator in apoptosis signaling pathway, was significantly affected in both Jurkat and U937 proteome profiles. The docking results suggested that 6-shogaol might bind well to eIF2α at Ser51 of the N-terminal domain. Immunoblotting data indicated that 6-shogaol induced apoptosis through a process involving dephosphorylation of eIF2α and caspase activation–dependent cleavage of eIF2α. Furthermore, 6-shogaol markedly inhibited tumor growth and induced apoptosis in U937 xenograft mouse model.

**Conclusion:**

The potent anti-leukemia activity of 6-shogaol found both *in vitro* and *in vivo* in our study make this compound a potential anti-tumor agent for hematologic malignancies.

## Background

In recent years, the use of natural dietary agents has become widely accepted as a realistic option for the treatment of malignant cancers because of their cost-effectiveness and wide safety margin. 6-Shogaol, a major pungent ingredient in ginger (*Zingiber officinale* Roscoe, Zingiberaceae), has attracted great attention due to its considerable pharmacologic effects including anti-cancer, anti-inflammatory, antioxidant, as well as antiemetic properties [1-4]. Evidences have revealed that 6-shogaol could induce cell death/apoptosis in a variety of cancer cells including human lung cancer, colorectal carcinoma, hepatocarcinoma, ovarian cancer and breast cancer cells [5-10].

Previous studies on the role of signaling cascades in 6-shogaol-related lethality have primarily focused on reactive oxygen species (ROS) production, activation of caspase, GADD 153 expression, tubulin polymerization, AKT/mTOR and matrix metalloproteinase 9 (MMP-9) expression [5,6,9,11]. The compound was also reported to inhibit breast cancer cell invasion by reducing MMP-9 expression via targeting the NF-*k*B activation cascade [9] or by inhibiting invade podium formation [12]. Our group and Gan et al. have found that 6-shogaol induced G2/M cell cycle arrest and apoptosis characterized by caspase 3 and PARP cleavage in HeLa and HCT116 cells [3,13]. Despite enormous efforts performed to investigate the anticancer activities of 6-shogaol, to date, the study on induction of apoptosis induced by 6-shogaol in human leukemia cells has not yet been explored, nor have the relationships between 6-shogaol-mediated anti-leukemic activity and cell signaling cascades been examined in depth.

Cell apoptosis involves two distinct pathways, the death receptor-initiated extrinsic pathway and the mitochondria-mediated intrinsic pathway [14,15]. Recent studies point to endoplasmic reticulum (ER) as a third subcellular compartment implicated in apoptotic execution [16,17]. Disruption of any function of ER causes ER stress and activates a cytoprotective signaling cascade called unfolded protein response (UPR) [18]. Different stimuli signal through several protein kinases to up-regulate the protein folding capacity of the ER through UPR induces the expression of ER chaperones such as GRP78/BiP [19]. Nowadays, the proteomic platform represents a powerful tool for profiling the comprehensive protein expression and investigation of the apoptotic mechanism of drugs. In particular the shotgun approach by label-free LC-CHIP Q-TOF MS/MS allows the qualitative and quantitative analysis of complex samples.

In the present study, we examined the apoptotic effects of 6-shogaol on three different leukemia cell lines and primary leukemia patients cells. A shotgun proteomics strategy based on LC-CHIP Q-TOF MS/MS was used to identify and quantify the differentially-expressed proteins of control and 6-shogaol-treated leukemia cells. Our results showed that cleavage of eIF2α and phospho-eIF2α by caspase activation may contribute to 6-shogaol-mediated cell death in human leukemia cells. Our *in vivo* results indicate that induction of apoptosis may contribute to 6-shogaol-mediated inhibitory effects on tumor growth of U937 xenograft mouse model. These findings provide a novel mechanistic basis for 6-shogaol as a leukemia treatment strategy.

## Results

### 6-Shogaol potently induced apoptosis in transformed and primary human leukemia cells, but not in normal bone marrow mononuclear cells

**Figure 1:**
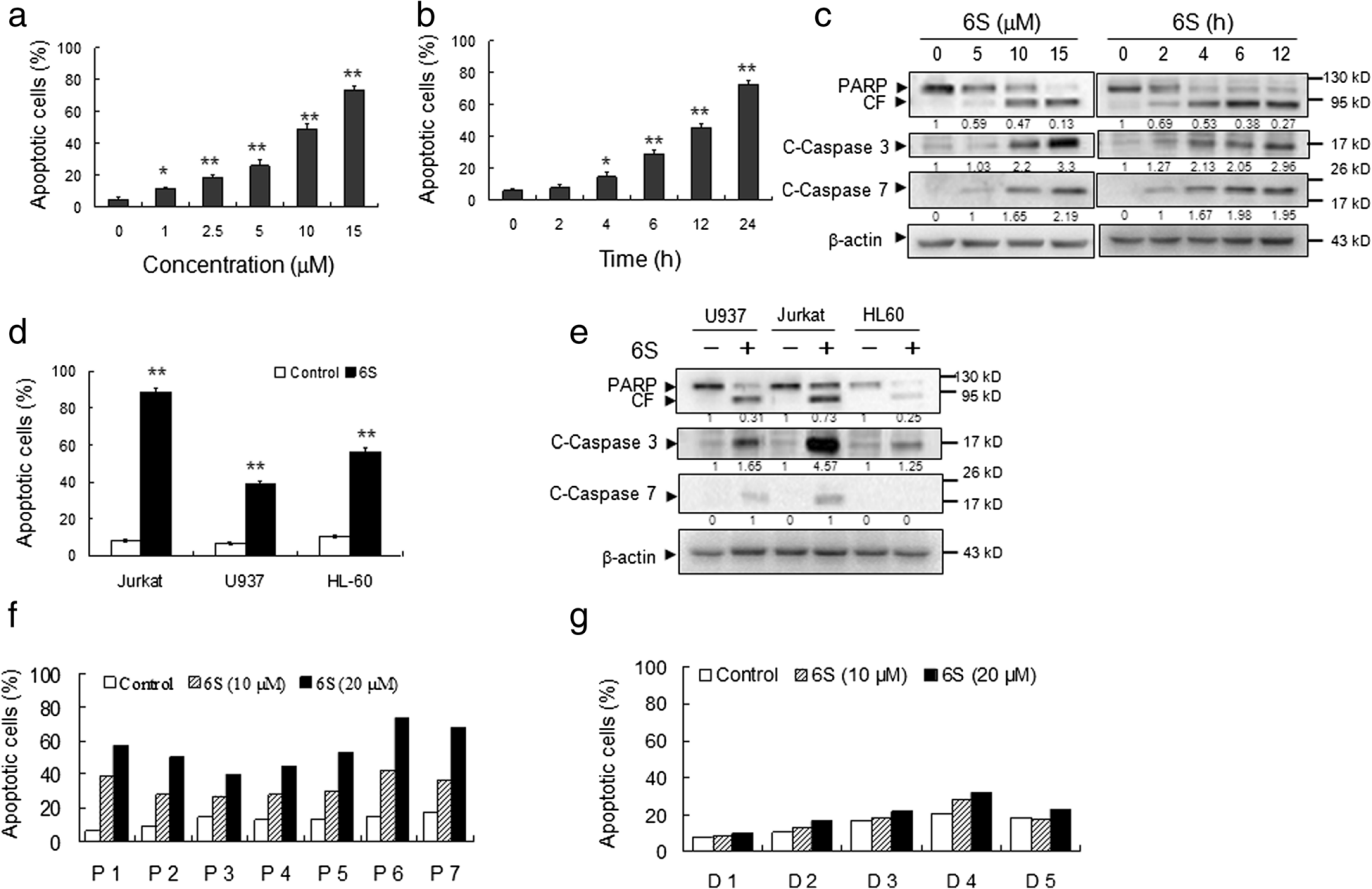
**6-Shogaol induces apoptosis in transformed and primary leukemia cells.** Cells apoptosis was determined using Annexin V/PI staining by flow cytometry. The values obtained from Annexin V/PI represent the mean±SD for three separate experiments. The difference were significant at *p < 0.05, **p < 0.01. **(a)** Jurkat cells were treated without or with various concentrations of 6-shogaol (6S) for 24 h. **(b)** Jurkat cells were treated without or with 15 μM for different time intervals as indicated. **(c)** Jurkat cell were treated without or with 6S for various concentrations or time intervals as indicated, total cellular extracts were prepared and subjected to western blot assay using antibodies against PARP, cleaved-caspase 3 (C-Caspase 3) and cleaved-caspase 7 (C-Caspase 7). (**d** and **e**) Jurkat, U937, and HL-60 cells were treated without or with 15 μM for 24 h, after which apoptosis was determined by flow cytometry. Total cellular extracts of protein were prepared and subjected to western blot using antibodies as indicated. **(f)** Mononuclear cells were isolated from the peripheral blood of 7 patients with leukemia (designated as P1–7), including 4 AML, 1 MM (P7), and 2 CLL patients (P3 and P4). Cells were then treated without or with 10 and 20 μM 6S for 24 hours. **(g)** Mononuclear cells were isolated from the peripheral blood of 5 healthy donors and incubated with 0, 10 and 20 μM of 6S for 24 hours.

Flow cytometry analysis revealed that treating Jurkat cells with 2.5 and 5 μM 6-shogaol for 24 h resulted in a moderate increase in apoptosis. These events became apparent at 10 μM and very extensive at 15 μM concentrations (Figure 1a). A time-course study of cells exposed to 15 μM 6-shogaol revealed a moderate increase in apoptosis as early as 6 h after drug exposure. These events became apparent after 12 h of drug exposure and reached near-maximal levels after 24 h of drug exposure (Figure 1b). Consistent with these findings, the same 6-shogaol concentrations and exposure intervals caused cleavage/activation of caspase-3 and caspase-7, as well as PARP degradation (Figure 1c).

To determine whether these events were restricted to myeloid leukemia cells, parallel studies were performed in other leukemia cell lines including U937 and HL-60 leukemia cells. These cells exhibited apoptotic effects of 6-shogaol similar to those observed in Jurkat cells (Figure 1d). Also, U937 and HL-60 cells caused comparable degrees of caspase-7 and caspase-3 activation and PARP degradation (Figure 1e).

To determine whether 6-shogaol could also trigger apoptosis in primary human leukemia cells, primary leukemia cells isolated from 7 leukemia patients were treated without or with 10 and 20 μM 6-shogaol for 24 h, after which apoptosis were determined by Annexin V/PI staining and flow cytometry. Exposure of cells to 6-shogaol resulted in pronounced increase in apoptosis in primary leukemia peripheral blood mononuclear cells (Figure 1f). In contract, the 6-shogaol regimen had no or little effect on apoptosis in normal bone marrow mononuclear cells (Figure 1g). Together, these findings indicate that 6-shogaol selectively kills transformed and primary human leukemia cells but not normal hematopoietic cells.

### Proteins alternation of leukemia cells in response to 6-shogaol treatment by LC-CHIP Q-TOF MS/MS

To get insights into the mechanism of apoptosis induced by 6-shogaol, an integrated proteomic-bioinformatics platform was used to investigate the global protein profiles of control and 6-shogaol-treated leukemia cells. In order to highlight the main proteome alterations in leukemia cells in response to 6-shogaol exposure, we generated protein expression profiles of two cell lines (Jurkat and U937) by a label-free shotgun proteomic approach after 12 h treatment with vehicle control (0.1% DMSO) or 15 μM 6-shogaol. The average peptide spectral intensity was used as a standard to normalize and compare the relative protein abundance in control and 6-shogaol treated cells [20,21]. More than 800 proteins were identified in our experiments. The identification of proteins with remarkable differences (up- or down-regulated over 2.0-fold) in Jurkat and U937 cells were shown in Additional file 1: Table S1 and Additional file 2: Table S2. The regulated proteins were listed by their protein name, accession number of SWISSPROT, abbreviations, MW/pI and fold change.

**Figure 2:**
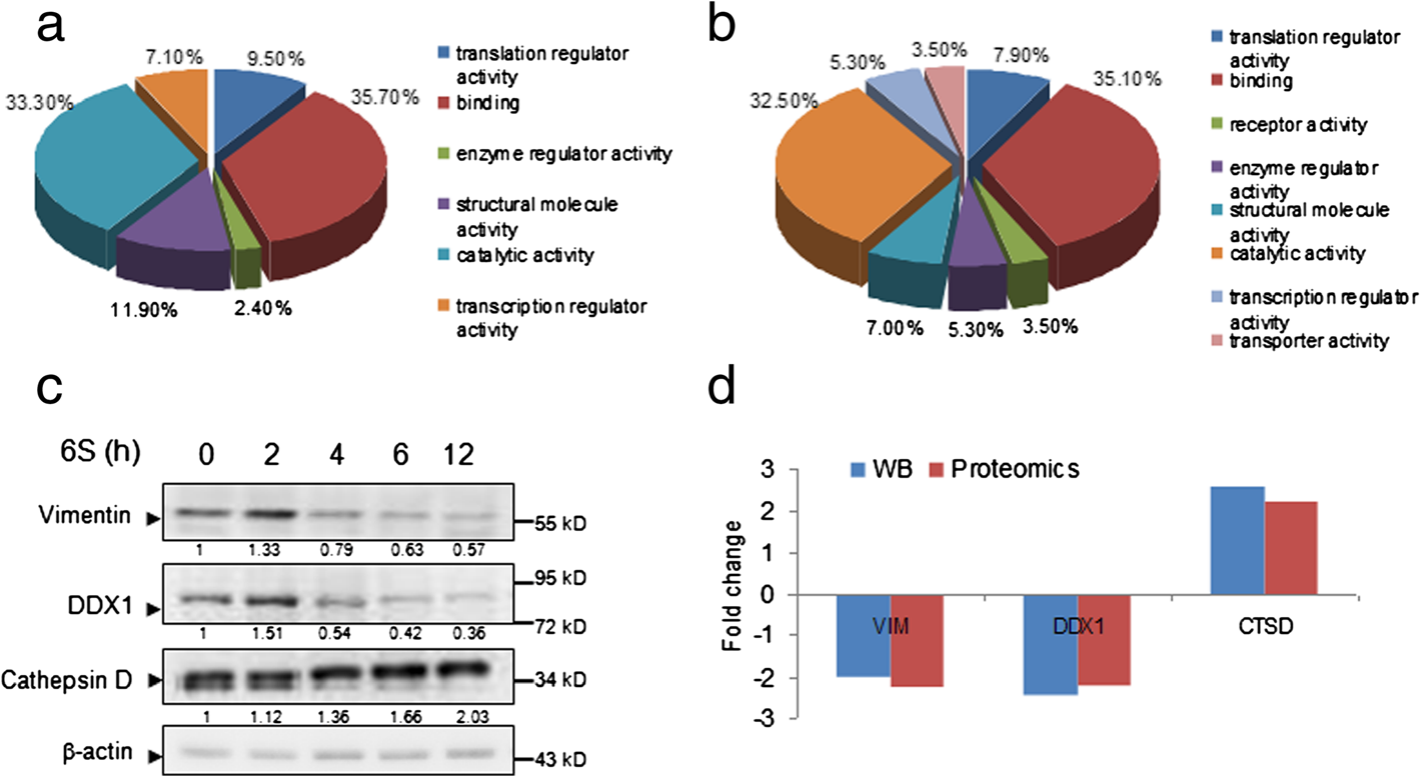
**Functional classification and validation of the differentially expressed proteins.** The changed proteins identified by LC-CHIP Q-TOF MS/MS for Jurkat **(a)** and U937 cells **(b)** were characterized according to their molecular functions by PANTHER Classification System. **(c)** Jurkat cells were treated with or without 6S (15 μM) for 12 h, and whole-cell lysates were obtained and subjected to western blot analysis using antibodies against Vimentin, DDX1, Cathepsin D and β-actin. Each blot is the representative result of three independent experiments. **(d)** The comparison of fold changes between proteins identified by mass spectrometry and proteins validated by western blot.

In the present study, all significantly modulated proteins were functionally categorized using the PANTHER Classification System (http://pantherdb.org) (As indicated in Figure 2a and 2b), the majority of differentially expressed proteins identified were in the categories of binding, catalytic activity, structural molecule activity, enzyme regulator activity and transcription regulator activity in both Jurkat and U937 cells. We also note that some differentially expressed proteins in the categories of receptor activity and transporter activity were only observed U937 cells.

To validate the proteome data, we used Western blot to assess the expression of three proteins (Vimentin, DDX1 and Cathepsin D) with higher fold change values, which were randomly selected from the list of 33 candidates based on their biologic interest, molecular weight, and antibody availability (Figure 2c). The data matched well with the differences exhibited in the proteome analysis (Figure 2d), which demonstrated the reliability of the proteomic analysis.

**Figure 3:**
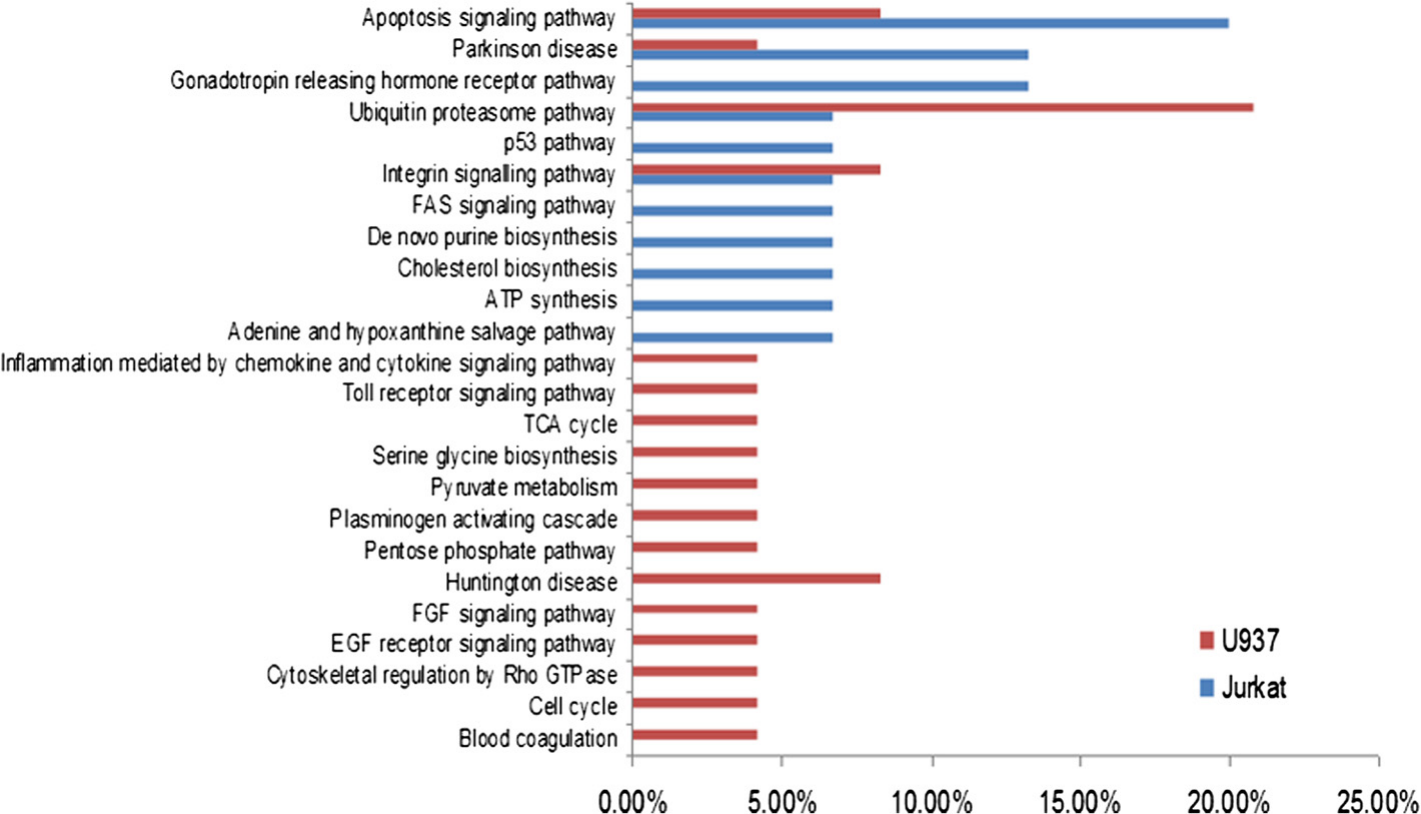
**Biological pathways associated with modulated proteins identified in U937 and Jurkat cells by proteomic analysis.** Both Jurkat and U937 cells were characterized according to their biological pathway by bioinformatics tool PANTHER Classification System. Percentage values shown as red bars indicate the number of U937 cell modulated proteins annotated to the respective GO biological pathway term divided by the number of all modulated proteins. The blue bars indicate the corresponding ratios for the reference data set in Jurkat cells. All biological pathways shown are with a *p*-value < 0.05.

To further understand the biological pathway involved in 6-shogaol regulated proteins, the PANTHER Classification System was used to categorize these proteins according to their biological processes. The results demonstrated that the 6-shogaol-regulated proteins could be classified into 24 pathways (Figure 3). Among which, four pathways including apoptosis, Parkinson, ubiquitin proteasome and integrin signaling were found to be involved in both Jurkat and U937 cell lines after 6-shogaol treatment. More differentially expressed proteins were found to be associated with the apoptosis signaling pathway only in 6-shogaol-treated Jurkat cells. While in 6-shogaol-treated U937 cells, more significantly modulated proteins were involved in the ubiquitin proteasome pathway. Also, three proteins, including GRP78/BiP, CYCS and EIF2S1, were found to be involved in the regulation of apoptosis in Jurkat cells, while EIF2S1 and AIFM1 were associated with apoptosis in U937 cell line.

### PERK-eIF2α cross-talk involved in 6-shogaol regulated apoptotic proteins

**Figure 4:**
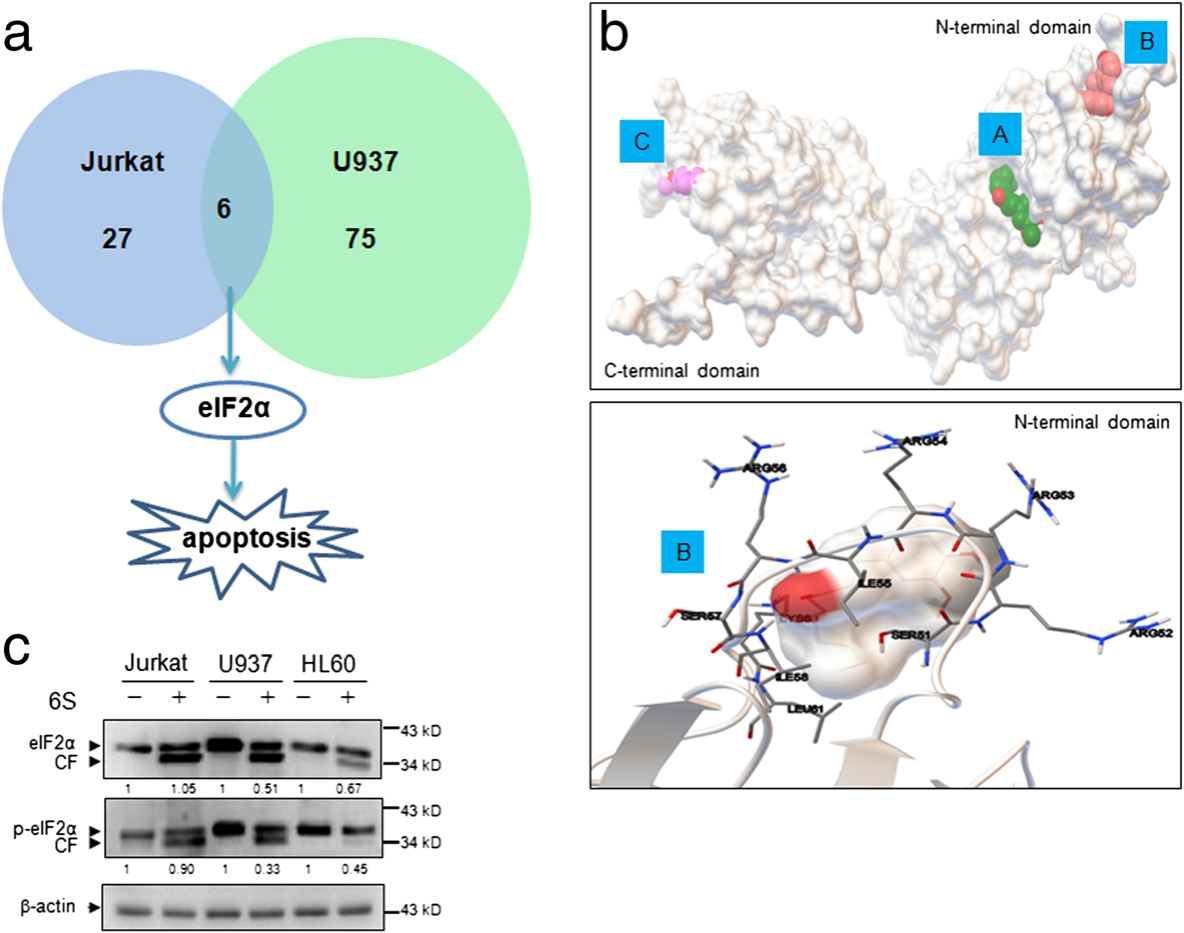
**Molecular docking analyses and validation by immunoblotting. (a)** Venn diagram depicting a comparison between proteins identified in Jurkat and U937 cell lines. Six proteins including eIF2α, a key regulator in apoptosis signaling pathway, were found to be altered in both cell lines. **(b)** The potential ligand-binding sites in eIF2α were analyzed and the most probable binding region are labeled with A, B and C. The protein is represented with cartoon model (up). 6S might bind to protein eIF2α at residue Ser51 of the N-terminal domain (down). The protein structure is displayed in ribbon model. **(c)** Validation of the docking results by three leukemia cell lines Jurkat, U937 and HL-60. Cells treated with or without 6S (15 μM) for 12 h were subjected to western blot analysis using antibodies against eIF2α and p-eIF2α (Ser 51). β-actin was used as reference. Each blot is the representative result of three independent experiments.

In the proteomics analyses, a total of 33 and 81 significantly modulated proteins were identified in 6-shogaol-treated Jurkat and U937 cell lines, respectively. Among them, six proteins including CTSD, EIF2S1, SSRP1, ILF3, GANAB and NONO (Additional file 1: Table S1 and Additional file 2: Table S2) were found to be altered in both cell lines (Figure 4a). EIF2S1, a key regulator in apoptosis signaling pathway (Figure 4a), may play a critical role in 6-shogaol-mediated lethality in leukemia cells.

To explore the interaction effect of 6-shogaol molecule to EIF2S1 (eIF2α), an in silico molecular docking study was performed. We docked 6-shogaol with the two main parts of eIF2α, C-terminal domain and N-terminal domain, separately [22]. Only those regions with binding energy < -5.0 kcal/mol were chosen as the “Potential Targets” [23]. As shown in Figure 4b (up), the potential binding sites may be present in the region A, B and C of eIF2α, since their binding energy was -6.02, -5.57 and -5.18 kcal/mol, respectively. It has been shown that, eIF2α contains the regulatory phosphorylation site, and a serine at position 51 in eIF2α is associated with the function of apoptosis [24]. Interestingly, serine at position 51 was found to be located in the pocket of region B in N-terminal domain (Figure 4b (down)). The docking results indicated that 6-shogaol might bind well to protein eIF2α at residue Ser51 of the N-terminal domain. To validate the interaction of 6-shogaol and the protein eIF2α, immunoblotting assay of eIF2α and phospho-eIF2α (Ser 51) in Jurkat, U937 and HL-60 cell lines was applied. The results indicated that exposure of cells to 6-shogaol resulted in reduction in levels of eIF2α and the production of its cleavage fraction (Figure 4c). The levels of phospho-eIF2α were also decreased and cleaved form of phospho-eIF2α was observed after treating cells with 6-shogaol. Such findings suggest that cleavage and dephosphorylation of eIF2α may contribute to 6-shogaol-mediated apoptosis in leukemia cells.

### Dephosphorylation and cleavage of eIF2α are required for 6-shogaol-induced apoptosis

**Figure 5:**
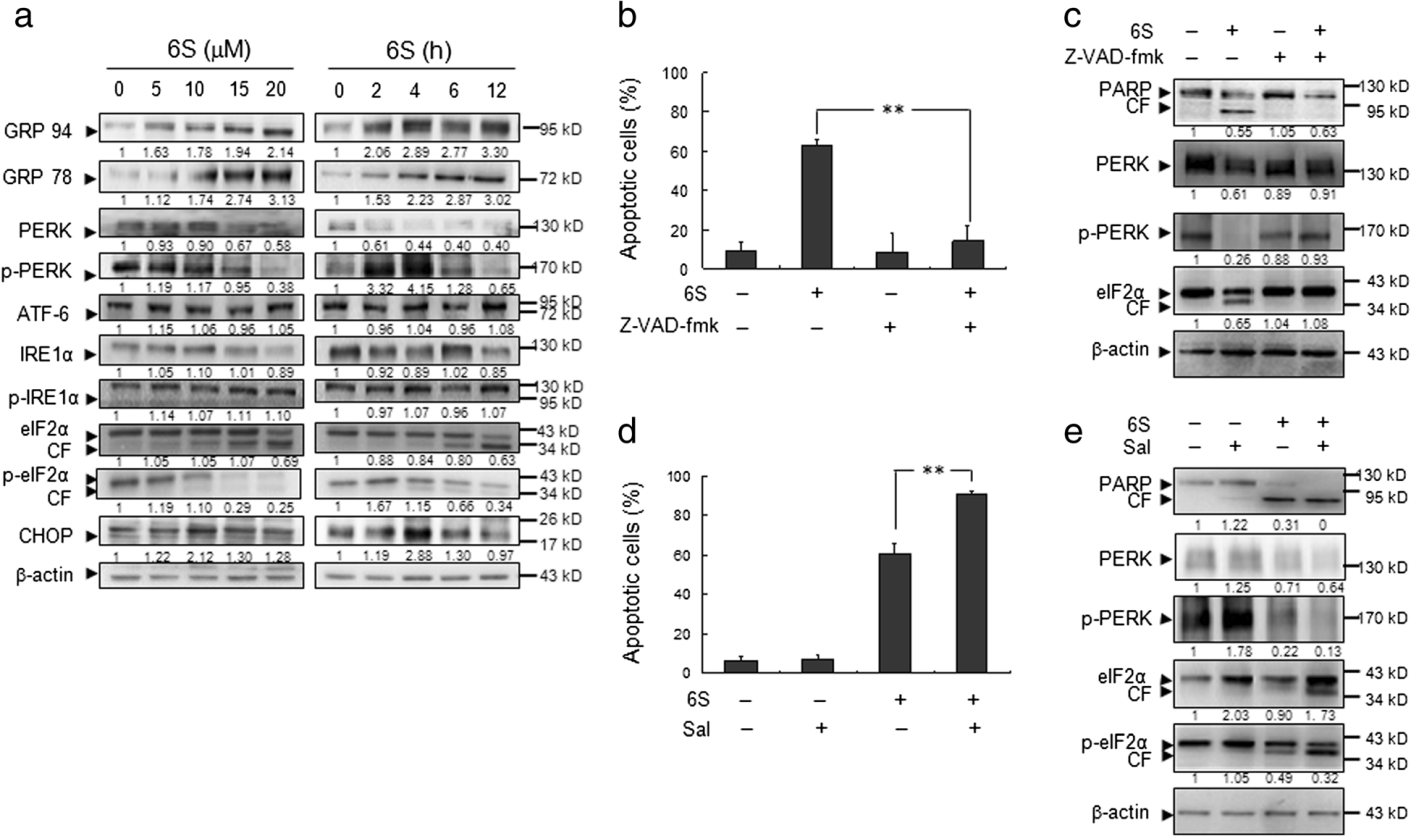
**Analysis eIF2α associated ER stress pathway related proteins in the effect of 6S induced apoptosis in Jurkat cells. (a)** Jurkat cells were treated with 15 μM of 6S for 0, 2, 4, 6, and 12 h or treated with various concentrations of 6S as indicated for 6 h. The total cellular extracts were subjected to western blot assay. β-actin was used as a loading control. Each blot is the representative result of three independent experiments. **(b)** Jurkat cells were pretreated with the caspase inhibitor Z-VAD-fmk (20 μM) for 1 h followed by treatment with 15 μM 6S for 12 h. Apoptosis was determined using flow cytometry. The graph shown represents the mean ± SD. for three separate experiments. The difference were significant at **p < 0.01. **(c)** Jurkat cells were treated with vehicle (DMSO 0.1%, v/v) alone or 6S (15 μM) or Z-VAD-fmk (20 μM) or the combination of 6S and Z-VAD-fmk for 12 h, western blot assay showed that Z-VAD-fmk markedly reduce 6S-induced apoptosis. Each blot is the representative result of three independent experiments. **(d)** Jurkat cells were treated with vehicle (DMSO 0.1%, v/v) alone or 6S (15 μM) or Sal (5 μM) or the combination of 6S and Sal for 12 h, cells were stained with Annexin V/PI, and the percentage of apoptotic cells was determined using flow cytometry. The graph shown represents mean ± SD for three separate experiments. The difference were significant at **p < 0.01. **(e)** Jurkat cells were treated with vehicle (DMSO 0.1%, v/v) alone or 6S (15 μM) or Sal (5 μM) or the combination of 6S and Sal for 12 h. Western blot assay showed that Sal potentiates 6S to induce Jurkat cells apoptosis through maintain the hyperphosphorylated state of eIF2α.

Protein alterations including protein synthesis, folding and chaperones related to ER stress promoted us to further investigate the role of ER stress pathway in 6-shogaol-induced apoptosis. Since UPR is an important genomic response to ER stress [25], the effects of 6-shogaol were examined in relation to UPR. Treatment of cells with 6-shogaol resulted in marked increase in levels of UPR targets GRP78/Bip and GRP94 in dose- and time-dependent manners (Figure 5a). Modest increase in levels of phospho-PERK and phospho-eIF2α after 2 h and 4 h of drug exposure was observed, and their normal forms were decreased after 6 h and 12 h of drug exposure (Figure 5a). Interestingly the cleaved forms of eIF2α and phospho-eIF2α were noted during the late time period of 6-shogaol treatment (Figure 5a). Similarly, the levels of GADD153/CHOP were increased at early time points of 6-shogaol exposure and then decreased at late time points of drug exposure (Figure 5a). In contrast, 6-shogaol had little or no effect on expression of ATF6, IRE1 and phospho-IRE1. Taken together, these findings demonstrate that PERK-eIF2α related ER stress pathway could play an important role in 6-shogaol-induced apoptosis in leukemia cells.

### 6-Shogaol-induced cleavage of eIF2α is dependent on caspase activation

During apoptosis, a class of cysteine proteases called caspases act as effectors of the cell death programme [26]. One mechanism by which caspases promote apoptosis is through cleavage and subsequent activation of protein kinases [27]. To observe whether 6-shogaol-induced cleavage of eIF2α and phospho-eIF2α is dependent on caspase activation, the pan-caspase inhibitor Z-VAD-fmk was used. Addition of Z-VAD-fmk blocked 6-shogaol-induced cell apoptosis and PARP degradation (Figure 5b and 5c). Interestingly, cleavage of eIF2α and dephosphorylation of PERK were inhibited by pretreatment with the caspase inhibitor Z-VAD-fmk (Figure 5c). Such findings indicate that 6-shogaol-mediated caspase activation may be involved in 6-shogaol-induced cleavage of eIF2α and dephosphorylation of PERK.

### Salubrinal synergizes with 6-shogaol to induce apoptosis through cleavage of eIF2α

Recent studies showed that eIF2α phosphorylation is required for cell survival, and inhibition of eIF2α phosphorylation enhanced cell death [28,29]. Salubrinal selectively blocks dephosphorylation of eIF2α and protects cells against ER stress-mediated apoptosis [30,31]. Thus we tested whether salubrinal could protect Jurkat cells against 6-shogaol-induced apoptosis. At a dose of 5 μM, salubrinal had no effect on apoptosis (Figure 5d), despite inducing eIF2α phosphorylation (Figure 5e). Unexpectedly, co-administration of salubrinal significantly enhanced 6-shogaol-mediated apoptosis (Figure 5d). As mentioned above, treating with salubrinal alone resulted in increased phosphorylation of eIF2α at 12 h. However, combined treatment of cells with 6-shogaol and salubrinal for 12 h did not further enhance eIF2α phosphorylation but resulted in pronounced cleavage of eIF2α. Such findings are consistent with the above results that 6-shogaol-mediated cleavage of eIF2α is dependent on caspase activation.

### 6-Shogaol inhibits tumor growth of U937 xenograft mouse model by striking induction of apoptosis

**Figure 6:**
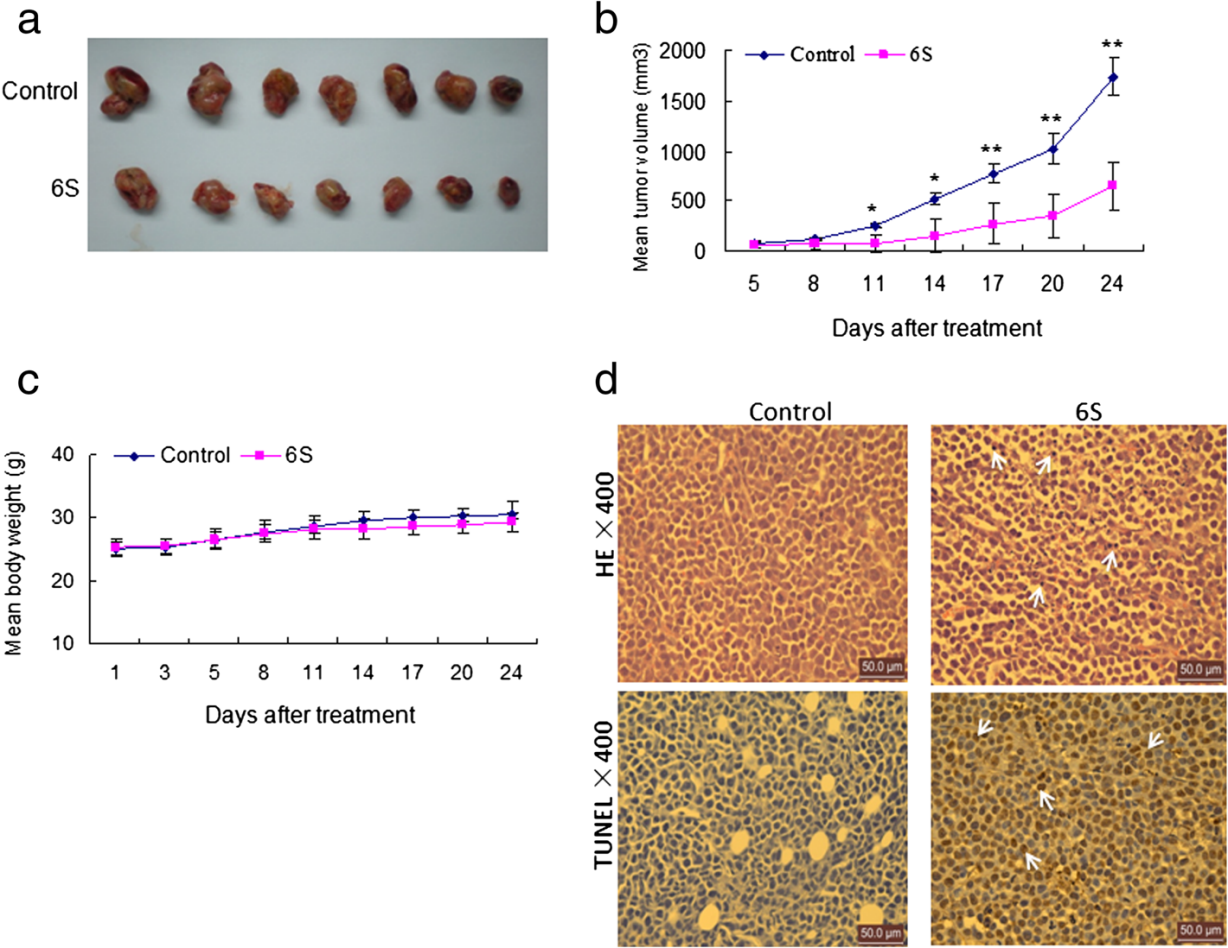
**6S markedly inhibits tumor growth and induces apoptosis in the xenograft animal model.** Five- to six-week-old NOD/SCID nude mice were injected subcutaneously with 2.5 × 10^6^ U937 cells into the right flank. From the fifth day, mice were randomized into a control group (7 mice per group) and treated group (7 mice per group, 6S of 50 mg/kg/day). Intraperitoneal administration of 6S and tumor volume assessment were conducted as described in the method of murine model. The 6S significantly inhibits tumor growth **(a)**, which is further confirmed by the tumor volume size **(b)**. Data are means ± SD. Values of tumor volume for 6S treatment groups were significantly decreased compared with those for the control group by Student’s t-test; *p < 0.05, **p < 0.01. The change of body weight is shown in **(c)**. Compared with the control group, TUNEL (× 400) assay shows increased numbers of apoptotic cells in the 6S treatment group **(d)**. Tumors were obtained from animals 20 days after drug exposure. Tumors were fixed and stained with hemtoxylin and eosin (H&E) stain to examine tumor cell morphology, using TUNEL assay to determine apoptosis.

The ability of 6-shogaol in killing human leukemia cells *in vitro* led us to evaluate its antileukemic activity *in vivo*. Of the leukemia cell lines tested, only the subcutaneous inoculation of U937 cells into NOD/SCID nude mice resulted in a tumor formation at the site of injection in mice (Figure 6a). Therefore, xenograft of human U937 cells was used in the present study. As shown in Figure 6b, treatment of mice with 50 mg/kg 6-shogaol resulted in a modest but significant suppression of tumor growth 11 days and 14 days following drug exposure (*p* < 0.05 vs vehicle control). These events became more apparent 17 days and 20 days and very extensive 24 days after drug exposure (*p* < 0.01 vs vehicle control) (Figure 6b). However, no significant changes in weight or other signs of potential toxicity were observed during the treatment with 6-shogaol (Figure 6c). We then examined the morphological changes and induction of apoptosis in tumor section of U937 xenografts using H&E staining and TUNEL assay. The sections of U937 xenografts from mice treated with 6-shogaol exhibited a reduced number of cancer cells, with signs of necrosis with infiltration of inflammatory cells and fibrosis (Figure 6d, top panels). Exposure to 6-shogaol resulted in a striking induction of apoptosis in tumor cells, with signs of numerous dark brown colored apoptotic cells (Figure 6d, bottom panels). Such findings suggest that 6-shogaol-mediated antileukemic activity *in vivo* is associated with induction of apoptosis.

## Discussion

In the present study, we demonstrate that 6-shogaol selectively induces apoptosis in diverse human leukemia cell lines as well as in primary human AML blast cells in dose- and time-dependent manners. However, 6-shogaol displayed less toxicity on normal human peripheral blood mononuclear cells, suggesting it may serve as a potentially valuable candidate for cancer chemotherapy.

For a comprehensive analysis of the molecular targets of 6-shogaol, we used the label free proteomics scheme by LC-CHIP Q-TOF MS/MS to identify the differentially expressed proteins in Jurkat and U937 leukemia cells after exposure to 6-shogaol. A total of 34 proteins whose expressions were significantly changed (over two folds) under 6-shogaol treatment were identified and quantified (see Additional file 1: Table S1 and Additional file 2: Table S2). Briefly, based on the protein function analysis, these 34 proteins could be generally classified into four categories: (i) protein folding and transcription; (ii) metabolism; (iii) cell death; and (iv) cell cytoskeleton structure. It must be noted that some proteins may have multiple functions and play roles in more than one pathway.

**Figure 7:**
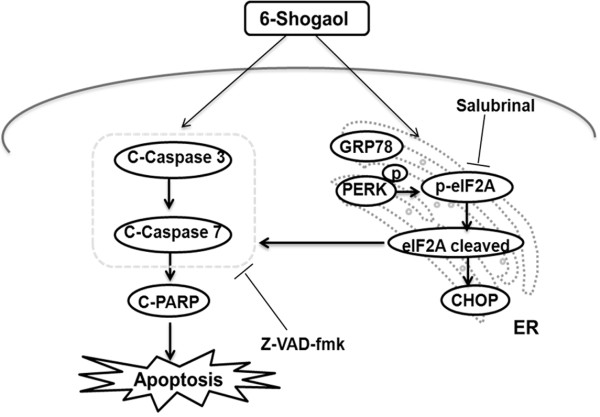
Schematic representation of the mechanisms of 6-shogaol in inducing apoptosis in human leukemia cells.

We have been interested in the mechanisms by which Jurkat cells recognize stress signals and regulate programs of gene expression designed to induce apoptosis. Central to cellular stress responses is a family of protein kinases that phosphorylate the α-subunit of eukaryotic initiation factor-2 (eIF2α). EIF2α phosphorylation by upstream kinases, like PERK or GCN2, can induce cell growth arrest or apoptosis in response to ER stress [32,33]. In cell apoptosis, the role of eIF2α phosphorylation may vary dependent on the cell type and apoptotic stimulus utilized [34]. Survival and resistance to chemotherapy are due to induction of eIF2α phosphorylation [35]. Otherwise, very intense eIF2α phosphorylation can activate programmed cell death. In the present report, we demonstrate that 6-shogaol induces phosphorylation of PERK and eIF2α at the early time points of drug exposure. EIF2α phosphorylation is inhibited at the late stage during apoptosis induced by 6-shogaol. Interestingly, the cleavage of eIF2α and phospho-eIF2α was induced in various leukemia cell lines during apoptosis induced by 6-shogaol. It has been reported that eIF2α is a target for cleavage by caspases upon induction of apoptosis in etoposide-treated cells [34]. Only caspase-3 was capable of eIF2α cleavage, which contributes to regulation of apoptosis. Consistent with these results, our findings demonstrate that caspases activation could contribute to cleavage of eIF2α during 6-shogaol-induced apoptosis based on the following findings: (i) 6-shogaol induces the cleavage of eIF2α and phospho-eIF2α at late time points; (ii) Inhibition of caspase activation by Z-VAD-fmk blocked 6-shogaol-mediated cleavage of eIF2α; (iii) Inhibition of caspase activation by Z-VAD-fmk also blocked 6-shogaol-induced apoptosis (Figure 7). Such findings suggest that the cleavage of eIF2α by caspase activation could contribute to inhibition or alteration of protein synthesis during the late stages of apoptosis induced by 6-shogaol.

It has been shown that salubrinal, a selective inhibitor of cellular complexes that dephosphorylate eIF2α, protects cells against ER stress-mediated apoptosis [31]. In our report, salubrinal treatment alone had no effect on apoptosis in leukemia cells despite inducing eIF2α phosphorylation. Unexpectedly, combined treatment of 6-shogaol with salubrinal for 12 h did not further enhance eIF2α phosphorylation but resulted in pronounced cleavage of eIF2α. These results were consistent with the previous reports that combination of bortezomib with salubrinal on RPMI 8226 or U266B1 cells resulted in pronounced cleavage of eIF2a and apoptosis [36]. This suggests that the combination therapy using 6-shogaol and salubrinal may be mostly suited for the treatment of leukemia.

Our previous study has shown that 6-shogaol can inhibit tumor growth of human hepatocellular xenografts through the induction of apoptosis [7]. Little is known, however, about inhibitory effects of 6-shogaol on tumor growth of human leukemia xenograft model. The results from *in vivo* studies demonstrated that 6-shogaol administration significantly inhibited the tumor growth of U937 xenograft without causing side effects to the mice. To understand whether the apoptotic mechanism *in vitro* is identical to those *in vivo*, we next examined apoptosis in tumor sections using TUNEL staining. A substantial increase of TUNEL-positive cells was detected in the 6-shogaol-treated group compared with the control group, which provides clear evidence for apoptosis in 6-shogaol-treated U937 xenograft mice. To the best of our knowledge, this is the first report that describes an effective extrapolation of the *in vitro* apoptosis-inducing effects of 6-shogaol on human leukemia cells to the *in vivo* situation.

## Conclusion

In summary, the present findings indicate that 6-shogaol effectively induces cell apoptosis in transformed and primary human leukemia cells, as well as in leukemia xenografts. This effect occurs in association with the cleavage of eIF2α during 6-shogaol-induced apoptosis, which is dependent on caspase activation. The potent anti-leukemia activity of 6-shogaol found both *in vitro* and *in vivo* in our study along with the novel mode of action make this compound a potential anti-tumor or prevent-tumor agent for hematologic malignancies. In addition, this work also identifies the cleavage of eIF2α as a potential biomarker of 6-shogaol-induced apoptosis. Further efforts are warranted to elucidate the mechanisms by which 6-shogaol induces the cleavage of eIF2α and to identify other possible factors that contribute to 6-shogaol-induced cell apoptosis. This study could provide a better understanding of how this compound exerts its antitumor activity *in vivo* and aid in developing this compound either alone or in combination with established chemotherapeutic agents to treat leukemia and potentially other hematologic malignancies.

## Methods

### Cells and reagents

Human acute T cell leukemia Jurkat, human histiocytic lymphoma U937 and human acute promyelocytic leukemia HL-60 cells were obtained from the American Type Culture Collection (Bethesda, MD, USA). Cells of Jurkat and U937 were maintained in RPMI-1640 medium with 10% heat-inactivated fetal bovine serum (FBS) in a humid atmosphere of 5% CO_2_ at 37°C. Cells of HL-60 were cultured in IMDM medium, supplemented with 20% FBS, the other conditions were the same as U937 and Jurkat. Fresh leukemia mononuclear cells from peripheral blood of seven leukemia (4 AML, 1 MM and 2 CLL according to FAB classification system) patients and five healthy donors were enriched by Ficoll separation. Informed consent was obtained according to institutional guidelines. Mononuclear cells were suspended in RPMI 1640 medium containing 10% FBS at a density of approximately 6–8 × 10^5^/mL for treatment.

6-Shogaol was isolated from *Z. officinale* in our laboratory [37] and its purity was not less than 98% detected using HPLC. Chemical regents, except for specially noted, were from Sigma (St. Louis, USA). Antibodies against PARP, PERK, CHOP, eIF2α, phosphor-eIF2α (Ser51; p-eIF2α), IRE1α, cleaved caspase-3,-7, and GRP78/BiP were obtained from Cell signaling Technology (Beverly, MA, USA). Phosphor-PERK (p-PERK) was purchased from Santa Cruz Biotechnology (CA, USA). Antibodies against ATF-6, phospho-IRE1α (S724; p-IRE1α), Vimentin, Cathepsin D, and DDX1 were from Abcam Biotechnology (Cambridge, UK). Salubrinal, a specific inhibitor of eIF2α-dephosphorylation, was purchased from Alexis (USA). Pan-caspase inhibitor of Z-VAD-fmk was from Beyotime Biotechnology (Beyotime, China). β-actin antibody, horseradish peroxidase-conjugated goat anti-mouse IgG, goat anti-rabbit IgG and rabbit anti-goat IgG were obtained from Bioss Biotechnology (Bioss, China).

### Apoptosis analysis

Apoptosis in cells were measured after treatment without or with 6-shogaol for various concentrations or time intervals as indicated. The cells were harvest, washed twice with ice-cold PBS and then determined with Annexin V-FITC Apoptosis Detection Kit (BD PharMingen, USA) by the manufacturer’s protocol as reported previously [38]. Analyses were applied on a FACS auto flow cytometer (BD Biosciences; Mountain View, CA). Both early apoptotic and late apoptotic cells were calculated in cell death determinations. Each experiment was performed in triplicate.

### Western blot

Cells were lysed in 200 μL WB&IP lysis buffer (1% Triton X-100) including 1 mM PMSF (Beyotime, China). Protein extracts (50 μg) were loaded onto a 6-15% polyacrylamide gel containing SDS, electrophoresed and transferred to a 0.22 μm nitrocellulose membrane (PALL, USA). The membranes were blocked with 5% non-fat dried milk in Tris-buffered saline/0.1% Tween 20 (TBST) and incubated overnight at 4°C with the appropriate primary antibody. The blots were washed with TBST three times and then probed with HRP-conjugated secondary antibodies for 2 h at room temperature. The immune complexes were visualized using a chemiluminescence phototope-horseradish peroxidase kit (Pierce, USA) as previously reported [38]. β-actin was used to ensure equivalent loading of whole cell protein. All the data were confirmed by three individual experiments.

### Shotgun proteomic analysis

The protein preparation, LC-CHIP Q-TOF MS/MS analysis and data processing were carried out as previously described [39]. Briefly, 50 μg preparation proteins were separated by SDS-PAGE. Then the PAGE was stained with Coomassie brilliant blue G-250 and cut into slices. Before MS analysis, the gel was destained and dehydrated. Then the proteins were digested with trypsin (10 ng/μL) and 40 mM ammonium bicarbonate/acetonitrile (9:1, *v*/*v*) at 37°C water bath overnight. After digestion, the peptides were extracted with solution containing 50% acetonitrile and 5% formic acid. The digested peptides were then concentrated and dried by speed vac to get lyophilized peptides. HPLC-CHIP (Agilent 1200 Series HPLC) was used to enrich and fractionate the resuspension peptide solution. Agilent 6520 ESI Q-TOF Mass Spectrometer adopted CHIP cube as ion source. A total of 1.0 μL sample (200 ng) was injected into the enrich column to desalt and then analyzed online through MS^*n*^ after isocratic eluted and gradient eluted by enrich column and separate column, respectively. Samples of each condition were run at least in triplicate. LC-MS and MS/MS data were processed by Spectrum Mill MS Proteomics Workbench (Rev A.03.03.078). Protein identification was obtained through the database of UniProtKB/SWISS-PROT especially for species of Homo sapiens. The value of peptide spectral intensity (or peptide chromatographic peak intensity, which means that MS precursor extracted ion chromatograms intensity for peptides that make up the same protein.) was from the analyzed data of MS and MS/MS. The MS/MS data files for processing were selected through the Spectrum Mill Data Extractor program, which extracts high-quality experimental fragmentation spectra from raw MS/MS data files. The screen parameters for data search were performed as previously described [39].

### Bioinformatics analysis

Modulated proteins identified by proteomic analysis were further analysed by the PANTHER (Protein Analysis THrough Evolutionary Relationships), a unique resource that possible to classifies genes or proteins by their molecular functions or pathways on the basis of published papers and by evolutionary relationships (version 7.2, http://www.pantherdb.org) [40]. The list of UniProt Accession from each protein was uploaded against the reference Homo sapiens dataset to summarize the molecular functional and biological process.

### Molecular docking study

To test the binding potency of 6-shogaol to protein eIF2α, an excellent in silico protein-ligand docking software for small ligand docking simulation, AutoDock 4.2 program was employed [41]. A series of steps were applied according to the standard protocols [42]: (1) Structure files of the target proteins were downloaded from PDB.org in the protein data bank; (2) Unnecessary substructures and water molecules were removed; (3) Addition of hydrogen atoms; (4) Calculation of the charges; (5) Run 100 times to give docked conformations; (6) Optimization of the positional, conformational, and orientational space of the ligand. Lamarckian genetic algorithm (LGA) was employed for each simulation process. The interaction figures were generated and the results of docking were recorded with binding energy and bonded residue.

### Murine model

Athymic nude mice (NOD/SCID, 5–6 weeks old) were obtained from the Shanghai Laboratory Animal Center (Shanghai, China). All animal studies were performed according to protocols approved by the Institutional Animal Care and Use Committee (IACUC) of the China Pharmaceutical University. After acclimatized to their new environment for 1 week, the mice were injected subcutaneously with U937 cells (2.5 × 10^6^/0.2 ml/mouse) into the right flank (day 0). Mice were randomized into two groups of 7 mice/group and 6-shogaol was dissolved in 0.1% DMSO and 10% polyoxyethylene castor oil. Three days after tumor inoculation, the treatment group received 6-shogaol (50 mg/kg per day for 20 days) and the control group received an equal volume of solvent control. The tumor volume (V) was measured every three days and calculated using the formula V = 0.5 × a × b^2^, where ‘a’ and ‘b’ are the length and width of the tumor, respectively. At the termination of the experiment, mice were killed at 24 h after the last administration of compound. The tumors were excised, fixed in formaldehyde and further processed for paraffin embedding.

### Detection of apoptosis by TUNEL in tumor tissue sections

The apoptosis cells in the tissues were detected using the TUNEL detection kit (*In situ* cell death detection kit-POD system, Roche). Briefly, the tumor tissues of paraffin embedded specimens were dewaxed in xylene and rehydrated with decreasing concentrations of ethanol [43]. Afterwards, DNA fragmentation was detected according to the manufacturer’s instructions. For quantification, three different fields were counted under light microscopy and at least 500 cells were enumerated in each field. All experiments were performed in duplicate [44].

### Statistical analysis

For analysis of data, values were presented as mean ± SD for the independent experiments. Statistical differences were determined by non-paired Student’s two-tailed *t* test and *p* < 0.05 was considered statistically significant.

## Abbreviations

C-caspase 3: Cleaved-caspase 3; C-caspase 7: Cleaved-caspase 7; eIF2α: Eukaryotic translation initiation factor 2 alpha; ER: Endoplasmic reticulum; FBS: Fetal bovine serum; MMP-9: Matrix metalloproteinase 9; PANTHER: Protein analysis through evolutionary relationships; ROS: Reactive oxygen species; 6S: 6-Shogaol; TBST: Tris-buffered saline/0.1% Tween 20; UPR: Unfolded protein response.

## Competing interests

The authors declare no competing interests.

## Authors’ contributions

QL participated in study design, performing experiments, and drafting of manuscript. Y-BP participated in study design and data analysis. PZ was involved performing experiments, acquisition of data and statistical analysis. L-WQ participated in study design and preparation the sample. MZ performed the molecular docking study. NG participated in study design and manuscript editing. E-HL and PL were involved in conception and design of study, data preparation and analysis, manuscript revisions. All authors read and approved the final version of manuscript.

## Supplementary Material

Additional file 1: Table S1 Identification of significantly changed proteins in 6-shogaol treated Jurkat cells using LC-CHIP Q-TOF MS/MS. Click here for file

Additional file 2: Table S2 Identification of significantly changed proteins in 6-shogaol treated U937 cells using LC-CHIP Q-TOF MS/MS. Click here for file
