# Supplementary material for: 6-Shogaol induces apoptosis in human leukemia cells through a process involving caspase-mediated cleavage of eIF2α
Source: Mol Cancer. 2013 Nov 12;12:135. doi: 10.1186/1476-4598-12-135 (PMC4176122; doi:10.1186/1476-4598-12-135)
Supplement: Additional file 1: Table S1 — Identification of significantly changed proteins in 6-shogaol treated Jurkat cells using LC-CHIP Q-TOF MS/MS. [file 1476-4598-12-135-S1.docx]

**Table S1** Identification of significantly changed proteins in 6-shogaol treated Jurkat cells using LC-CHIP Q-TOF MS/MS.

| No. | Protein Name | Gene Name | Swiss Prot | MW | PI | Fold Change |
| --- | --- | --- | --- | --- | --- | --- |
| 1 | 26S protease regulatory subunit 8 | PSMC5 | P62195 | 45626.30 | 7.11 | 3.20 |
| 2 | 40S ribosomal protein S17 | RPS17 | P08708 | 15550.20 | 9.85 | 2.38 |
| 3 | 78 kDa glucose-regulated protein precursor | GRP78/BiP | P11021 | 72333.30 | 5.07 | 2.10 |
| 4 | Adenine phosphoribosyltransferase | APRT | P07741 | 19607.90 | 5.78 | 2.20 |
| 5 | Annexin A5 | ANXA5 | P08758 | 35936.90 | 2.78 | 2.78 |
| 6 | ATP-dependent RNA helicase DDX1 | DDX1 | Q92499 | 82432.60 | 6.81 | -2.22 |
| 7 | C-1-tetrahydrofolate synthase, cytoplasmic | MTHFD1 | P11586 | 101559.80 | 6.89 | -2.50 |
| 8 | **Cathepsin D precursor** | CTSD | P07339 | 44552.50 | 6.10 | 2.20 |
| 9 | Chromobox protein homolog 3 | CBX3 | Q13185 | 20811.50 | 5.22 | 3.37 |
| 10 | Cytochrome c | CYCS | P99999 | 11748.80 | 9.59 | 2.66 |
| 11 | Cytosol aminopeptidase | LAP3 | P28838 | 56166.70 | 8.03 | 3.52 |
| 12 | DNA replication licensing factor MCM6 | MCM6 | Q14566 | 92889.80 | 5.29 | -4.17 |
| 13 | Eukaryotic translation elongation factor 1 epsilon-1 | EEF1E1 | O43324 | 19810.70 | 8.55 | 2.00 |
| 14 | **Eukaryotic translation initiation factor 2 subunit 1** | EIF2S1 | P05198 | 51109.80 | 8.66 | -2.00 |
| 15 | Eukaryotic translation initiation factor 3 subunit 4 | EIF3G | O75821 | 35611.20 | 5.87 | -2.00 |
| 16 | **FACT complex subunit SSRP1** | SSRP1 | Q08945 | 81075.20 | 6.45 | -2.78 |
| 17 | Filamin-B | FLNB | O75369 | 278196.50 | 5.49 | 3.60 |
| 18 | GMP synthase [glutamine-hydrolyzing] | GMPS | P49915 | 76715.80 | 6.42 | -3.13 |
| 19 | High mobility group protein B3 | HMGB3 | O15347 | 22980.10 | 8.48 | -2.86 |
| 20 | Histidine triad nucleotide-binding protein 2 | HINT2 | Q9BX68 | 17161.80 | 9.20 | 2.50 |
| 21 | **Interleukin enhancer-binding factor 3** | ILF3 | Q12906 | 95338.90 | 8.85 | -2.04 |
| 22 | Mesoderm development candidate 2 | MESDC2 | Q14696 | 26076.80 | 7.60 | 4.74 |
| 23 | **Neutral alpha-glucosidase AB precursor** | GANAB | Q14697 | 106874.50 | 5.73 | 5.34 |
| 24 | **Non-POU domain-containing octamer-binding protein** | NONO | Q15233 | 54231.90 | 9.01 | -4.55 |
| 25 | Poly [ADP-ribose] polymerase 1 | PARP1 | P09874 | 113084.40 | 8.99 | -2.08 |
| 26 | Protein mago nashi homolog 2 | MAGOHB | Q96A72 | 17275.90 | 5.96 | 2.00 |
| 27 | Prothymosin alpha [Contains: Thymosin alpha-1] | PTMA | P06454 | 12203.00 | 3.69 | -3.85 |
| 28 | Ras GTPase-activating protein-binding protein 1 | G3BP1 | Q13283 | 52164.50 | 5.37 | -2.70 |
| 29 | Septin-1 | 1-Sep | Q8WYJ6 | 41971.00 | 5.56 | -2.04 |
| 30 | Squalene synthetase | FDFT1 | P37268 | 48115.60 | 6.10 | -3.85 |
| 31 | Tubulin-specific chaperone A | TBCA | O75347 | 12854.90 | 5.25 | 2.20 |
| 32 | Vacuolar protein sorting-associated protein 29 | VPS29 | Q9UBQ0 | 20505.80 | 6.28 | 3.37 |
| 33 | Vimentin | VIM | P08670 | 53651.90 | 5.06 | -2.27 |
