# Supplementary material for: 6-Shogaol induces apoptosis in human leukemia cells through a process involving caspase-mediated cleavage of eIF2α
Source: Mol Cancer. 2013 Nov 12;12:135. doi: 10.1186/1476-4598-12-135 (PMC4176122; doi:10.1186/1476-4598-12-135)
Supplement: Additional file 2: Table S2 — Identification of significantly changed proteins in 6-shogaol treated U937 cells using LC-CHIP Q-TOF MS/MS. [file 1476-4598-12-135-S2.docx]

**Table S2** Identification of significantly changed proteins in 6-shogaol treated U937 cells using LC-CHIP Q-TOF MS/MS

| No. | Protein Name | Gene Name | Swiss Prot | MW | PI | Fold Change |
| --- | --- | --- | --- | --- | --- | --- |
| 1 | 0S ribosomal protein S19 | RPS19 | P39019 | 16060.6 | 10.31 | -3.99 |
| 2 | 26S protease regulatory subunit 7 | PSMC2 | P35998 | 48634.1 | 5.71 | -2.38 |
| 3 | 26S proteasome non-ATPase regulatory subunit 3 | PSMD3 | O43242 | 60977.9 | 8.47 | -2.38 |
| 4 | 26S proteasome non-ATPase regulatory subunit 6 | PSMD6 | Q15008 | 45531.6 | 5.45 | 4.90 |
| 5 | 40S ribosomal protein S11 | RPS11 | P62280 | 18430.8 | 10.31 | -2.64 |
| 6 | 60S ribosomal protein L10 | RPL10L | P27635 | 24604.1 | 10.11 | -2.37 |
| 7 | 60S ribosomal protein L17 | RPL17P9 | P18621 | 21397.2 | 10.18 | -2.27 |
| 8 | 60S ribosomal protein L19 | RPL19 | P84098 | 23466.1 | 11.48 | -2.33 |
| 9 | 6-phosphogluconate dehydrogenase, decarboxylating | PGD | P52209 | 53140.3 | 6.8 | 2.40 |
| 10 | Actin-related protein 2/3 complex subunit 2 | ARPC2 | O15144 | 34333.2 | 6.84 | -2.15 |
| 11 | ADP-ribosylation factor 1 | ARF3 | P84077 | 20696.9 | 6.31 | -2.23 |
| 12 | ADP-ribosylation factor-like protein 8B | ARL8B | Q9NVJ2 | 21539.2 | 8.67 | 2.63 |
| 13 | Alpha-2-macroglobulin receptor-associated protein precursor | LRPAP1 | P30533 | 41466.2 | 8.73 | -14.75 |
| 14 | Alpha-soluble NSF attachment protein | NAPSA | P54920 | 33232.9 | 5.23 | -2.39 |
| 15 | Annexin A1 | ANXA1 | P04083 | 38714.5 | 6.57 | 12.46 |
| 16 | Apoptosis-associated speck-like protein containing a CARD | PYCARD | Q9ULZ3 | 21626.9 | 5.95 | -2.17 |
| 17 | Apoptosis-inducing factor 1, mitochondrial precursor | AIFM1 | O95831 | 66900.9 | 9.04 | 2.77 |
| 18 | Basigin precursor | BSG | P35613 | 42200.6 | 5.39 | -2.51 |
| 19 | Caprin-1 | CAPRIN1 | Q14444 | 72752.1 | 4.76 | -8.96 |
| 20 | **Cathepsin D precursor** | CTSD | P07339 | 44552.5 | 6.1 | 3.81 |
| 21 | Citrate synthase, mitochondrial precursor | CS | O75390 | 51712.7 | 8.45 | 2.46 |
| 22 | D-3-phosphoglycerate dehydrogenase | PHGDH | O43175 | 56650.8 | 6.29 | 2.32 |
| 23 | DNA replication licensing factor MCM2 | MCM2 | P49736 | 101896.7 | 5.34 | -2.05 |
| 24 | DNA replication licensing factor MCM3 | MCM3 | P25205 | 90981.3 | 5.53 | -4.66 |
| 25 | Dolichyl-diphosphooligosaccharide--protein glycosyltransferase 67 kDa subunit precursor | RPN1 | P04843 | 68569.7 | 5.96 | 2.42 |
| 26 | Erlin-1 precursor | ERLIN1 | O75477 | 38926 | 7.67 | 5.43 |
| 27 | **Eukaryotic translation initiation factor 2 subunit 1** | EIF2S1 | P05198 | 36112.4 | 5.02 | -2.04 |
| 28 | Eukaryotic translation initiation factor 3 subunit 10 | EIF3A | Q14152 | 166570.2 | 6.38 | -2.39 |
| 29 | Eukaryotic translation initiation factor 3 subunit 6-interacting protein | EIF3L | Q9Y262 | 66727.3 | 5.93 | -2.52 |
| 30 | Eukaryotic translation initiation factor 3 subunit 9 | EIF3B | P55884 | 92492.1 | 4.89 | -3.41 |
| 31 | Eukaryotic translation initiation factor 4 gamma 1 | EIF4G1 | Q04637 | 175535.9 | 5.27 | -6.31 |
| 32 | Eukaryotic translation initiation factor 4H | EIF4H | Q15056 | 27385.2 | 6.67 | -2.27 |
| 33 | Eukaryotic translation initiation factor 5B | EIF5B | O60841 | 138800.4 | 5.38 | -3.95 |
| 34 | **FACT complex subunit SSRP1** | SSRP1 | Q08945 | 81075.2 | 6.45 | -13.99 |
| 35 | Fatty acid synthase | FASN | P49327 | 273401 | 5.99 | -2.60 |
| 36 | Fumarylacetoacetase | FAH | P16930 | 46374.6 | 6.46 | -2.14 |
| 37 | Glutathione transferase omega-1 | GSTO1 | P78417 | 27566 | 6.24 | 2.09 |
| 38 | HLA class I histocompatibility antigen, B-18 alpha chain precursor | B-3501 | P30466 | 40275.1 | 6.28 | -2.45 |
| 39 | HLA class I histocompatibility antigen, B-7 alpha chain precursor | B*15 | P01889 | 40460.3 | 5.57 | -2.33 |
| 40 | Hsc70-interacting protein | ST13 | P50502 | 41331.9 | 5.18 | -2.15 |
| 41 | Importin subunit beta-3 | IPO5 | O00410 | 123630.7 | 4.83 | -2.84 |
| 42 | I**nterleukin enhancer-binding factor 3** | ILF3 | Q12906 | 95338.9 | 8.85 | -2.59 |
| 43 | Lamin-B receptor | LBR | Q14739 | 70703.6 | 9.41 | -3.08 |
| 44 | Leucine-rich PPR motif-containing protein, mitochondrial precursor | LRPPRC | P42704 | 157905.9 | 5.81 | 2.90 |
| 45 | Liver carboxylesterase 1 precursor | CES1 | P23141 | 62521.4 | 6.15 | 2.03 |
| 46 | Lon protease homolog, mitochondrial precursor | LONP1 | P36776 | 106489.9 | 6.01 | 2.74 |
| 47 | **Neutral alpha-glucosidase AB precursor** | GANAB | Q14697 | 106874.5 | 5.73 | 4.84 |
| 48 | **Non-POU domain-containing octamer-binding protein** | NONO | Q15233 | 54231.9 | 9.01 | -3.92 |
| 49 | NSFL1 cofactor p47 | NSFL1C | Q9UNZ2 | 40573 | 4.99 | -3.71 |
| 50 | Nuclear autoantigenic sperm protein | NASP | P49321 | 85238.1 | 4.26 | -2.02 |
| 51 | Nuclear migration protein nudC | NUDC | Q9Y266 | 38243.1 | 5.27 | -2.10 |
| 52 | Nuclease sensitive element-binding protein 1 | YBX1 | P67809 | 35924.3 | 9.88 | -2.47 |
| 53 | Nucleolar RNA helicase 2 | DDX21 | Q9NR30 | 87344.8 | 9.32 | -2.62 |
| 54 | Phosphatidylethanolamine-binding protein 1 | PEBP1 | P30086 | 21056.9 | 7.01 | -2.01 |
| 55 | Plasminogen activator inhibitor 2 precursor | SERPINB2 | P05120 | 46596.5 | 5.46 | -2.83 |
| 56 | Plectin-1 | PLEC | Q15149 | 531735.2 | 5.73 | -2.66 |
| 57 | Polyadenylate-binding protein 1 | PABPC3 | P11940 | 70671.3 | 9.51 | -2.03 |
| 58 | Prohibitin | PHB | P35232 | 29804.2 | 5.57 | 2.16 |
| 59 | Proteasome subunit beta type-3 | PSMB3 | P49720 | 22949.1 | 6.14 | 2.20 |
| 60 | Proteasome subunit beta type-5 precursor | PSMB5 | P28074 | 22897 | 8.66 | -2.82 |
| 61 | Protein DJ-1 | PARK7 | Q99497 | 19891.2 | 6.33 | -2.21 |
| 62 | Protein FAM49B | FAM49B | Q9NUQ9 | 36748.2 | 5.76 | -2.49 |
| 63 | Protein KIAA1967 | KIAA1967 | Q8N163 | 102902.1 | 5.14 | -2.60 |
| 64 | Putative RNA-binding protein 3 | RBM3 | P98179 | 17170.4 | 8.86 | -3.45 |
| 65 | RNA-binding protein 8A | RBM8A | Q9Y5S9 | 19889.1 | 5.5 | -2.44 |
| 66 | RNA-binding protein FUS | FUS | P35637 | 53426.1 | 9.4 | -3.99 |
| 67 | RNA-binding protein Raly | RALY | Q9UKM9 | 32463.3 | 9.2 | -3.02 |
| 68 | Splicing factor U2AF 65 kDa subunit | U2AF2 | P26368 | 53501.3 | 9.19 | -2.53 |
| 69 | Splicing factor, arginine/serine-rich 6 | SRSF6 | Q13247 | 39587 | 11.42 | -9.83 |
| 70 | Superoxide dismutase [Mn], mitochondrial precursor | SOD2 | P04179 | 24722.2 | 8.34 | 2.13 |
| 71 | T-complex protein 1 subunit gamma | CCT3 | P49368 | 60534.3 | 6.1 | -2.12 |
| 72 | T-complex protein 1 subunit theta | CCT8 | P50990 | 59620.9 | 5.42 | -2.17 |
| 73 | T-complex protein 1 subunit zeta | CCT6A | P40227 | 58024.5 | 6.24 | -2.59 |
| 74 | Transcription intermediary factor 1-beta | TRIM28 | Q13263 | 88550.2 | 5.52 | -2.22 |
| 75 | Transmembrane and coiled-coil domain-containing protein 1 | TMCO1 | Q9UM00 | 21175.1 | 9.77 | 3.01 |
| 76 | Transmembrane emp24 domain-containing protein 9 precursor | TMED9 | Q9BVK6 | 25104.9 | 6.67 | 2.61 |
| 77 | Trifunctional enzyme subunit beta, mitochondrial precursor | HADHB | P55084 | 51294.8 | 9.44 | 2.46 |
| 78 | Tumor protein D54 | TPD52L2 | O43399 | 22237.8 | 5.26 | -3.69 |
| 79 | Ubiquitin-conjugating enzyme E2 N | UBE2N | P61088 | 17137.9 | 6.13 | -2.39 |
| 80 | Unc-112-related protein 2 | FERMT3 | Q86UX7 | 75953.2 | 6.52 | -3.53 |
| 81 | XTP3-transactivated gene A protein | DCTPP1 | Q9H773 | 18681 | 4.92 | -3.16 |
